# Application of urine C-peptide creatinine ratio in type 2 diabetic patients with different levels of renal function

**DOI:** 10.3389/fendo.2022.1052794

**Published:** 2022-11-17

**Authors:** Wan Zhou, Jie Li, Xiaojing Yuan, Wei Wang, Huanran Zhou, Haoqiang Zhang, Shandong Ye

**Affiliations:** ^1^ Department of Endocrinology, The First Affiliated Hospital of USTC, Division of Life Sciences and Medicine, University of Science and Technology of China, Hefei, China; ^2^ Anhui Provincial Hospital, Affiliated to Anhui Medical University, Hefei, China; ^3^ The First Affiliated Hospital of USTC, Division of Life Sciences and Medicine, University of Science and Technology of China, Hefei, China

**Keywords:** diabete, urinary C peptide/creatinine ratio, 24-hour urinary C-peptide, EGFR, C peptide

## Abstract

**Objective:**

This study aims to investigate the effect of single urine C peptide/creatinine (UCPCR) in assessing the islet β Cell function of type 2 diabetes mellitus (T2DM) patients with different renal function.

**Methods:**

A total of 85 T2DM patients were recruited in this study, all the patients were assigned to one group with estimated glomerular filtration rate (eGFR)≤60 ml·min^-1^·1.73 m^-2^ and another group complicated with eGFR>60 ml·min^-1^·1.73 m^-2^. Serum creatinine, urine creatinine, serum fasting C-peptide (FCP), fasting blood glucose (FBG), glycosylated hemoglobin (HbA1C) and 24-hour urinary C-peptide (24hUCP) were measured. The modified homeostasis model assessment-islet β cell function [HOMA-islet (CP-DM)], the modified homeostasis model assessment-insulin resistance [HOMA-IR(CP)] and UCPCR were calculated.

**Results:**

When compared with group eGFR ≤60 ml·min^-1^·1.73 m^-2^, the levels of UCPCR, FCP, the modified HOMA-IR(CP) and HOMA-islet (CP-DM) were promoted and the concentrations of HbA1C, FPG, creatinine were decreased in the patients of eGFR>60 ml·min^-1^·1.73 m^-2^ (*P*<0.05); FCP was uncorrelated with 24hUCP while associated with UCPCR in the patients of eGFR ≤ 60 ml·min^-1^·1.73 m^-2^; UCPCR was positively correlated with FCP and HOMA-IR(CP) in the T2DM patients with different levels of renal function; the cut-off (UCPCR ≤ 1.13 nmol/g) had 88.37% sensitivity and 95.24% specificity [95% confidence interval (CI):0.919-0.997] for identifying severe insulin deficiency in T2DM patients[area under the curve (AUC) 0.978].

**Conclusion:**

UCPCR can be used to evaluate islets β Cell function in T2DM patients with different renal function status.

## 1 Introduction

Diabetes is a chronic metabolic disease, and its prevalence is increasing at an alarming rate along with the population aging and lifestyle changes. The number of diabetes patients is projected to rise to about 592 million globally including about 143 million in China by 2035, which will bring a large economic burden on society (1). Type 2 diabetes mellitus (T2DM) with islet β-cell secretory dysfunction and insulin resistance as the major characteristics, accounts for around 95% of all cases of diabetes (2). Therefore, the evaluation of islet function in T2DM patients has gained clinical significance.

C-peptide (CP) is a peptide hormone containing 31 amino acids and is formed during the cleavage of insulin from proinsulin secreted by islet β-cells. CP can be measured in blood and urine and is not affected by exogenous insulin. The blood CP can reflect the instantaneous level at the time of blood collection. 60–69% of the blood CP is filtered through the glomerulus, and 80% is reabsorbed by the renal tubules and metabolized by the kidney (3). The daily output of CP is about 5% of the insulin secretion. In patients with normal renal function, 24-h urine CP (24-h UCP) reflects the average value of blood CP produced in a day, and its stability is better than that of blood CP (4). The radioimmunoassay is the commonly used method to detect 24-h UCP and can be used to evaluate the functional state of islet β-cells. Thus, it helps in the diagnosis, classification, and prognosis of diabetes (5). 24-h UCP can also be used to predict the postoperative pancreas remanent volume and the functional gastrointestinal tract reconstruction (6). However, it is inconvenient to collect 24-h UCP samples, and the accuracy of 24-h UCP is found to be poor in diabetes patients with moderate and severe renal damage (7). Therefore, there is an urgent need for a simple and convenient approach to evaluate the function of islet β-cells, which can eliminate the impact caused by renal damage. Single urine CP creatinine ratio (UCPCR) is a detection index that uses CP/creatinine (Cr) ratio to correct urine dilution. Previous studies have shown that UCPCR can be used to determine hepatocyte nuclear factor 1alpha (HNF1α) and HNF4α for distinguishing between maturity-onset diabetes of the young (MODY) and other types of diabetes (8, 9). Recent studies have shown that UCPCR can also be used to evaluate the function of islet β-cells in patients with T1DM and T2DM (10, 11). However, no study has been carried out to assess the application of UCPCR for the evaluation of islet β-cell function in patients with T2DM till now, especially in T2DM patients with differences in renal function. In this study, estimated glomerular filtration rate (eGFR), 24-h UCP, blood CP, and UCPCR were measured in T2DM patients to investigate the potential application of UCPCR in the evaluation of islet function in T2DM patients with different levels of renal function. The study can provide a basis for the application of UCPCR in the clinical practice of diabetes.

## 2 Materials and methods

### 2.1 Subjects

Considering the inconvenience of 24-h urine sample collection, the patients recruited in this study are all inpatients. Eighty-five T2DM patients including 56 males and 29 females, with an average age of 57.72 ± 4.43 years, who were hospitalized in the Department of Endocrinology from February 2020 to December 2021 were selected.

All subjects were given oral hypoglycemic drugs or exogenous insulin therapy except insulin secretagogues before admission. T2DM was diagnosed according to the World Health Organization (WHO) diagnostic criteria 1999. Exclusion criteria of this study include diabetes ketoacidosis, hyperglycemia and hypertonic state, severe infection, stress state, and severe liver dysfunction. Based on the eGFR, the patients were divided into two groups: eGFR≤ 60 mL·min-1·1.73 m-2 and eGFR>60 mL·min-1·1.73 m-2. All the subjects signed informed consent, and this study was conducted in accordance with the tenets of the World Medical Association’s Declaration of Helsinki and had been approved by the ethics committee of The First Affiliated Hospital of USTC.

### 2.2 Methods

#### 2.2.1 Specimen collection

The height, weight, age, gender, course of the disease, and other general information of all the subjects were recorded. Blood samples and single urine samples were collected in the morning after a 12-hour fast. Five mL of each morning urine sample was stored at -80°C and was used for the detection of urine creatinine (UCr) and UCP. After the single urine samples were collected, the rest of urine samples over 24 h beginning from 09:00 am on that day to 09:00 am on the next day were collected to measure 24-h UCP.

#### 2.2.2 Specimen detection

7600-120 automatic biochemical instrument (Hitachi, Tokyo, Japan) was used to detect fasting blood glucose (FPG), serum creatinine (SCr), blood urea nitrogen (BUN), and UCr. Hemoglobin A1c (HbAlc) was determined by high-pressure liquid chromatography (Primus Ultra, Ireland), whereas fasting insulin (Fins), fasting CP (FCP), 24-h UCP, and morning urine UCP were measured by chemiluminescence [The intra- and inter-assay coefficients of variability were 1.5% and 3.5%%, respectively] on an Atellica IM analyzer (Siemens Healthcare Diagnostics, Shanghai). The lower limit of the CP assay was 0.03 ng/ml, and for this analysis, all CP concentrations<0.03 ng/ml were recorded as 0.03 ng/ml. The upper limit was 30 ng/ml, and all CP concentrations>30 ng/ml were recorded as 30 ng/ml.

#### 2.2.3 Calculation of the indicators

eGFR was calculated by the modified form of the Modification of Diet in Renal Disease (MDRD) equation. The input parameters in the eGFR calculation software were gender, SCr value, and the age of the patient. The formula for calculating eGFR is given by eGFR (mL·min^-1^·1.73 m^-2^) = 30849 × SCr (umol/L)^-1.154^ × Age (years)^-0.203^ × Gender coefficient (male coefficient is 1, female coefficient is 0.742), and can be used to estimate eGFR of all the subjects. Instead of insulin, fasting C peptide was used to evaluate insulin resistance and islet function.

The modified homeostasis model assessment-islet β cells was calculated according to the formula [HOMA-islet (CP-DM)] = 0.27 × FCP (nmol/L)/[FPG (mmol/L) [3.5]. The insulin resistance index is assessed by the modified homeostasis model assessment-insulin resistance and expressed as, [HOMA-IR (CP)] = 1.5 + FPG (mmol/L) × FCP (nmol/L)/2800 (12). UCPCR is given by UCP/UCr and the body mass index (BMI) is calculated as weight (kg)/height (m^<xsp>2</xsp>^).

### 2.3 Statistical treatment

Statistical Product and Service Solutions (SPSS) 22.0 software was used to perform a statistical analysis of the data. The measured data were expressed as mean ± standard deviation (± s). The independent sample t-test was used for the comparison between groups. Multivariable logistic regression and pearson analysis was used for the correlation analysis. Receiver operating characteristic (ROC) curve were used to identify cut-off values of UCPCR for distinguishing islet cell function in T2DM patients. Statistical significance was accepted at *p* < 0.05.

## 3 Results

### 3.1 Comparison of clinical characteristics between the two groups

The average value of eGFR ≤ 60 mL·min^-1^·1.73 m^-2^ group was observed to be 48.79 ± 6.44 mL·min^-1^·1.73 m^-2^, while that of eGFR>60 mL·min^-1^·1.73 m^-2^ group was observed to be 90.26 ± 16.8 mL·min^-1^·1.73 m^-2^. Compared to T2DM patients with eGFR ≤ 60 mL·min^-1^·1.73 m^-2^, patients with eGFR>60 mL·min^-1^·1.73 m^-2^ were younger and had a shorter course of disease, lower HbA1c, FPG, SCr, UCr and higher 24-h UCP, UCP, UCPCR, FCP, HOMA- HOMA-islet (CP-DM), HOMA-IR (CP) (*p* < 0.05) (**Table 1**).

**Table 1:** Comparison of clinical parameters between the two groups of patients.

Characteristics	eGFR ≤ 60	eGFR>60	*t*	*P*
*n*	40	45	/	/
Age(y)	71.48 ± 4.4	65.2 ± 4.14	6.767	<0.001
Duration (y)	9.13 ± 3.26	5.78 ± 1.85	5.908	<0.001
BMI (kg/m2)	24.83 ± 2.34	25.25 ± 2.71	-0.77	0.443
HbA1C (%)	10.13 ± 1.89	8.51 ± 2.07	3.774	<0.001
FPG (mmol/L)	8.49 ± 1.65	7.79 ± 1.23	2.230	0.028
FCP (nmol/L)	0.20 ± 0.02	0.39 ± 0.05	-20.701	<0.001
24h UCP (nmol/L)	3.46 ± 0.99	6.61 ± 1.27	-12.599	<0.001
BUN (mmol/L)	5.90 ± 1.13	6.12 ± 1.47	-0.766	0.446
Cr (umol/L)	116.8 ± 11.95	70.63 ± 9.35	19.948	<0.001
eGFR	48.79 ± 6.44	90.26 ± 16.8	-14.671	<0.001
UCr (umol/L)	16348.79 ± 2610.88	9664.73 ± 1987.66	13.364	<0.001
UCP (nmol/L)	1.61 ± 0.25	1.87 ± 0.38	-3.707	<0.001
UCPCR (nmol/g)	0.89 ± 0.21	1.67 ± 0.41	-10.740	<0.001
HOMA-islet (CP-DM)	12.29 ± 6.25	27.51 ± 12.66	-6.892	<0.001
HOMA-IR (CP)	2.10 ± 0.13	2.57 ± 0.17	-14.276	<0.001

### 3.2 Results of multiple linear regression analysis

As depicted in **Table 2**, FCP in T2DM patients was used as the dependent variable, while 24h UCP and UCPCR were used as the independent variables. After adjusting for gender and age, multivariable logistic regression analysis showed that FCP was associated with both UCPCR and 24h UCP in T2DM patients with eGFR > 60 mL·min^-1^·1.73 m^-2^ while it was associated with UCPCR but uncorrelated with 24hUCP in the patients of eGFR ≤ 60 ml·min^-1^·1.73 m^-2^.

**Table 2:** Results of multiple linear regression analysis.

Groups	B	SE	t	*P*
GFR ≤ 60				
24h UCP	-0.005	0.004	-1.188	0.243
UCPCR	0.080	0.014	5.576	<0.001
GFR>60				
24h UCP	0.019	0.006	3.485	0.001
UCPCR	0.059	0.018	3.339	0.002

### 3.3 Correlation analysis between UCPCR and various indicators under different eGFR conditions

**Figure 1:**
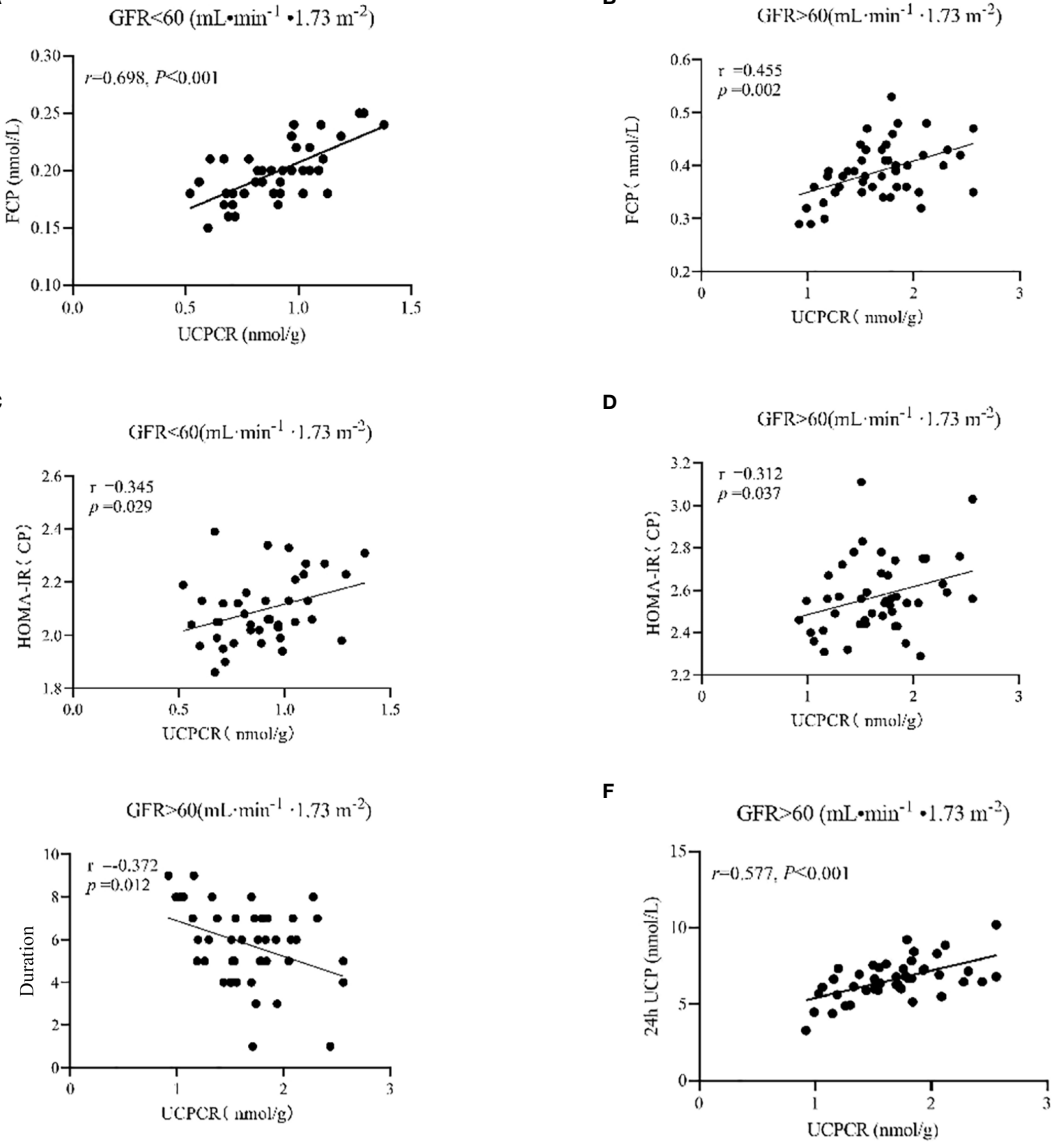
When eGFR ≤ 60 mL.min-1.1.73 m-2, UCPCR was positively correlated with FCP **(A)** and HOMA-IR (CP) **(C)**; When eGFR > 60 mL.min-1.1.73 m-2, UCPCR was positively correlated with FCP **(B)**, HOMA-IR(CP) **(D)** and 24-h UCP **(F)**; UCPCR was negatively correlated with duration **(E)**.

When eGFR > 60 mL·min^-1^·1.73 m^-2^, UCPCR was positively correlated with FCP (r = 0.455, *p* = 0.002), 24-h UCP (r = 0.577, *p<0.001*), and HOMA-IR(CP) (r = 0.312, *p* = 0.037), while it was negatively correlated with duration (r = -0.372, *p* = 0.012). When eGFR ≤ 60 mL·min^-1^·1.73 m^-2^, UCPCR was positively correlated with FCP (r = 0.698, *p<0.001*) and HOMA-IR(CP) (r = 0.345, *p* = 0.029) (**Table 3** and **Figure 1**).

**Table 3:** Correlation analysis between UCPCR and other indicators.

		Age(y)	Duration (y)	BMI(kg/m2)	HbA1C(%)	FPG(mmol/L)	FCP(nmol/L)	24h UCP(nmol/L)	HOMA-IR (CP)	HOMA-islet (CP-DM)
eGFR ≤ 60	*r*	0.154	-0.224	-0.288	0.128	-0.047	0.698	-0.052	0.345	0.290
	*P*	0.342	0.164	0.071	0.432	0.773	<0.001	0.750	0.029	0.069
eGFR>60	*r*	0.254	-0.372	0.148	-0.014	-0.106	0.455	0.577	0.312	0.166
	*P*	0.092	0.012	0.330	0.930	0.489	0.002	<0.001	0.037	0.275

When eGFR ≤ 60 mL·min-1·1.73 m-2, UCPCR was positively correlated with FCP (a) and HOMA-IR(CP) (c); When eGFR > 60 mL·min-1·1.73 m-2, UCPCR was positively correlated with FCP (b), HOMA-IR(CP) (d) and 24-h UCP (f);UCPCR was negatively correlated with duration (e).

### 3.4 ROC curve analysis for UCPCR in distinguishing the function of islet β cells in T2DM patients

**Figure 2:**
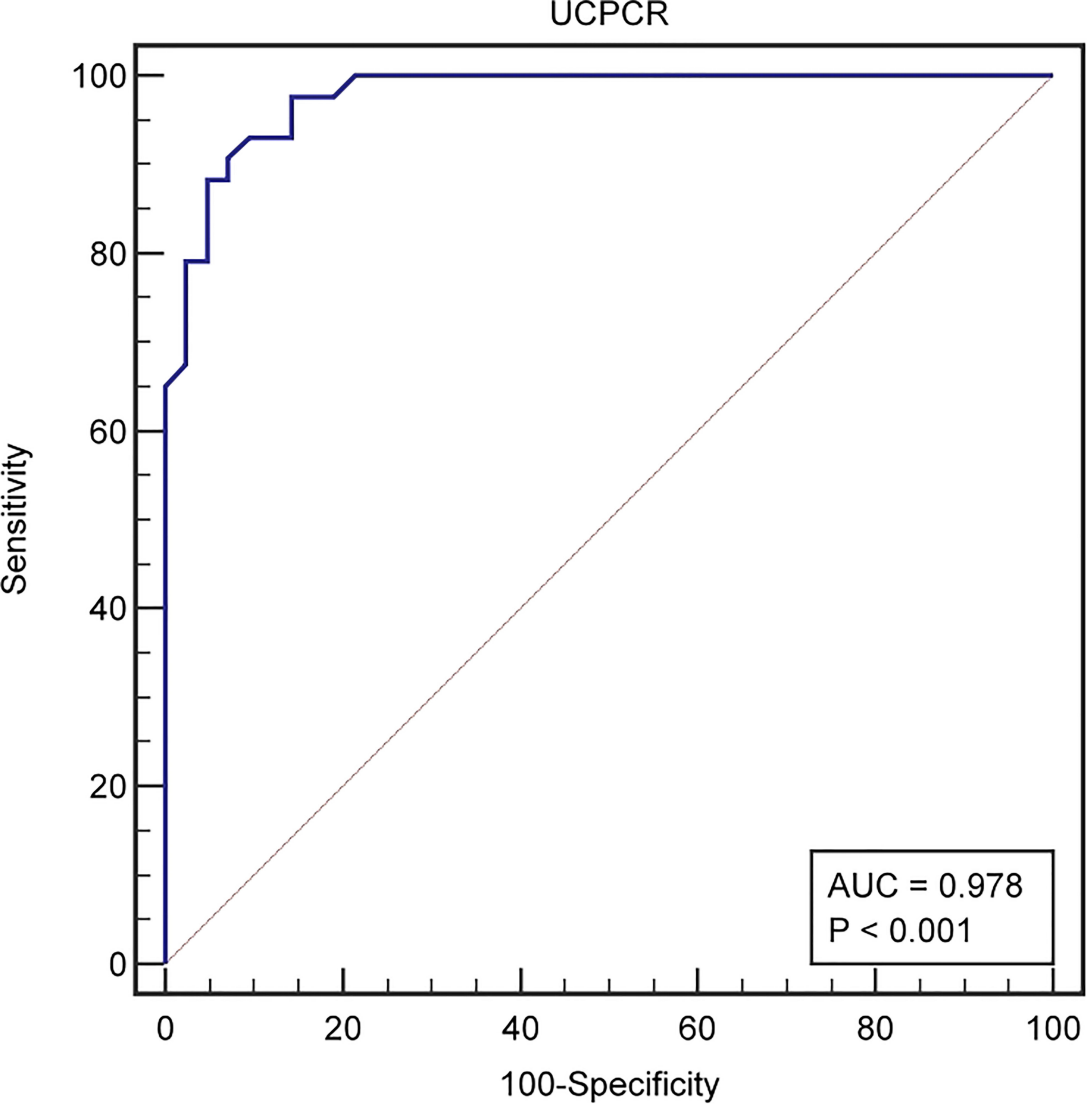
The ROC curve identified a cut-off UCPCR ≤1.13 nmol/g for discriminating poor islet function in T2DM patienets (AUC 0.978) with 88.37% sensitivity and 95.24% specificity.

UCPCR was well-correlated with FCP. FCP < 0.3mmol/l are suggestive of marked insulin deficiency (13). ROC curve were used to identify the cutoff of UCPCR that provided the optimal sensitivity and sensitivity for distinguishing the function of islet β cells in T2DM patients by the software MedCalc V15.2. UCPCR cut-off ≤1.13 nmol/g had the highest Youden index for detecting FCP < 0.3mmol/l and thus identifying severe insulin deficiency, with 88.37% sensitivity and 95.24% specificity (AUC, 0.978, 95% confidence interval (CI) (0.919–0.997), *P* < 0.001) (**Figure 2**).

## 4 Discussion

The evaluation of islet function includes the evaluation of insulin sensitivity and the secretion ability of islet β-cells which refers to the synthesis and secretion of insulin and peptides by β-cells. There are some indicators, e.g., hyperglycemic clamp technique, insulin secretion index, insulin resistance index, the area under the C-peptid curve, that can be used to evaluate the islet function. C-peptide is often used as a simple index to clinically evaluate the function of the islet, which is produced by the enzymatic cleavage of proinsulin, secreted in equal amounts as endogenous insulin, and mainly cleared by the kidney (14). Blood CP reflects the instantaneous CP level at the time of blood collection. As CP is easily degraded by prion enzymes, the sample should be immediately centrifuged after blood collection and sent for examination on ice. The stability of 24-h UCP is better than that of blood CP and can reflect the one-day average CP level in the subjects. Recent study have shown that 24-h UCP can be used for the evaluation of islet β-cell function and can indicate the blood CP level in case of normal renal function (15). Jamal et al. (16) found that the blood and UCP levels of T1DM patients were lower than those of control group, and there was a positive correlation between blood CP and 24-h UCP in each group. The 24-h UCP values can also be used to classify diabetes. A study by Nouran et al. found that the levels of blood CP and 24-h UCP in T1DM patients were significantly lower than those in the control group and T2DM group, indicating poor secretion of insulin from the islet β-cells in T1DM patients (17). There were no significant differences in 24-h UCP between patients with normal renal function and patients with mild and moderate renal insufficiency, and 24-h UCP was positively correlated with blood CP (18). However, 24-h UCP test has some limitations. For diabetes patients with moderate and severe renal damage, 24-h UCP cannot reflect the blood CP levels. Koskinen et al. (19) reported that blood CP and 24-h UCP values were significantly different in diabetes patients with moderate and severe renal damage, which indicated that the decreases of the clearance of CP by kidney result in the retention of CP when renal function is damaged. In this study, when compared with T2DM patients with mild renal damage, 24-h UCP and FCP decreased in patients with moderate and severe renal damage. The multivariable logistic regression indicated that FCP was associated with 24h UCP in T2DM patients with eGFR > 60 mL·min^-1^·1.73 m^-2^ while it had no correlation with 24hUCP in the patients of eGFR ≤ 60 ml·min^-1^·1.73 m^-2^, which is in accordance with the previous research findings. In addition, 24-h urine collection is cumbersome, and is not suitable for outpatients. If urine is diluted or concentrated, it can affect CP levels, thus limiting its wide clinical application.

UCPCR is a non-invasive outpatient diagnostic tool. Previous studies have shown that UCPCR can be used to distinguish between MODY and T2DM patients affected for more than 2 years (20). Recent studies have shown that UCPCR is a useful alternative to blood CP and 24-h UCP tests, and can better reflect the function of the islet. Other tests have some limitations such as the instability of blood CP and the difficulty in 24-h UCP sample retention, but UCPCR sample can be stored for at least 3 days at room temperature with the addition of boric acid preservative (21). The experiment adopted in this paper has little interference from proteinuria and naked eye hematuria, and has stable performance and high stability. Therefore, UCPCR is especially suitable for outpatients to monitor insulin secretion. It is easy to perform and is not affected by the location of sample collection. Elzahar et al. (22) reported that UCPCR is a simple, non-invasive and reliable marker, which can be used for the diagnosis of T2DM and T1DM in children. As a non-invasive marker, UCPCR can distinguish between T1DM and T2DM or monogenic diabetes (11, 23). UCPCR ≤ 0.20 nmol/mmol reflects the severe impairment of β-cell function in T2DM patients (24). In our study, the cut-off (UCPCR ≤ 1.13 nmol/g equals 0.13 nmol/mmol) for a differential diagnosis of T2DM is also helpful to identify patients who need insulin or secretagogue therapy. A study by Ryota et al. (25) observed that UCPCR was significantly lower in the insulin-treated patients than in the insulin-untreated patients and, so it was applicable for differentiating T2DM from T1DM. In a previous study conducted by our research group, UCPCR had a good correlation with blood CP and 24-h UCP in T2DM patients and can be used as a practical indicator for insulin secretion in T2DM patients (26).

Urine C-peptide is easily affected by urine volume, renal function, etc. As a product of muscle tissue metabolism, UCr is filtered through the glomerulus, and its daily excretion in urine is stable. When compared with 24-h UCP, UCPCR can overcome the differences caused by urine dilution by measuring UCP and the corresponding UCr in a single morning urine sample and calculating their ratio. This method can overcome the influence of renal function on the UCP value, thus avoiding the interferences caused by urinary tract infection, neurogenic bladder and other factors. It can be used to evaluate the islet β-cell function in diabetes patients with renal insufficiency. At present, only a few studies evaluate the impact of differences in renal function on UCPCR. McDonald et al. reported that UCPCR can be used to replace 24-h UCP in patients with renal insufficiency to evaluate the secretory function of islet β-cells (27). Bowan et al. (28) observed that UCPCR was positively correlated with serum CP in diabetic patients with moderate renal damage (eGFR ≤ 60 mL·min^-1^·1.73 m^-2^). This study observed that in T2DM patients with eGFR ≤ 60 mL·min^-1^·1.73 m^-2^, FCP was uncorrelated with 24-h UCP, but it was still correlated positively with UCPCR. The correlation analysis indicated that UCPCR in patients with differences in renal function was positively correlated with FCP, implying that UCPCR can be used for evaluation of islet β-cell function in T2DM patients with differences in renal function. In addition, this study observed improved insulin resistance and functional indices. Insulin can be replaced by fasting CP, which can better indicate the correlation between CP and UCPCR. In this study, UCPCR was positively correlated with HOMA-IR (CP) in patients with differences in renal function. These findings are consistent with the research results of Oram et al. (29), indicating that UCPCR also reflects the insulin resistance levels of T2DM patients with differences in renal function.

The novelty of this study is to analyze and compare UCPCR, 24-h UCP, FCP, HOMA-IR(CP) and HOMA-islet (CP-DM) in T2DM patients with differences in renal function. It is evident that UCPCR is not affected by the state of the renal function and hence can be used to detect the islet function and insulin resistance levels of diabetes patients with differences in renal function. UCPCR can better evaluate the islet β-cell function in T2DM patients. This provides a basis for the application of UCPCR in the clinical diagnosis and treatment of diabetes. However, this study also has some limitations, such as the small sample size. Therefore, there is a need to carry out prospective research with a large sample size in the future. In addition, future studies also need to determine the cut-off point value of UCPCR in identifying differences in renal function.

There were some limitations in our study. Firstly, it was a single-center and hospital-based study, the sample size was relatively small. In addition, only one urine sample was collected from each patient, while postprandial C peptide and urine C peptide had not been collected. Further studies are necessary to extend the validity of our findings.

## 5 Conclusion

Briefly, UCPCR has great repeatability and the samples collected for this test are easy to retain. This test can reflect the islet function in T2DM patients with differences in renal function, which can significantly increase its clinical application and popularity.

## Data availability statement

The raw data supporting the conclusions of this article will be made available by the authors, without undue reservation.

## Ethics statement

All the subjects signed informed consent, and this study was conducted in accordance with the tenets of the World Medical Association’s Declaration of Helsinki and had been approved by the ethics committee of The First Affiliated Hospital of USTC. The patients/participants provided their written informed consent to participate in this study.

## Author contributions

WZ designed the research, analyzed the data, and wrote the manuscript. JL and XY collected the data. HQZ and HRZ analyzed the data. SY and WW reviewed and edited the manuscript. WZ is the guarantor of this work. All authors contributed to the article and approved the submitted version.

## Funding

This work was supported by the Project of National Natural Science Foundation of China (Grant No. 82270863 to WZ).

## Conflict of interest

The authors declare that the research was conducted in the absence of any commercial or financial relationships that could be construed as a potential conflict of interest.

## Publisher’s Note

All claims expressed in this article are solely those of the authors and do not necessarily represent those of their affiliated organizations, or those of the publisher, the editors and the reviewers. Any product that may be evaluated in this article, or claim that may be made by its manufacturer, is not guaranteed or endorsed by the publisher.
